# Socio-demographic characteristics and their relation to medical service consumption among elderly in Israel during the COVID-19 lockdown in 2020 as compared to the corresponding period in 2019

**DOI:** 10.1371/journal.pone.0278893

**Published:** 2022-12-15

**Authors:** Ohad Shaked, Liat Korn, Yair Shapiro, Gideon Koren, Avi Zigdon

**Affiliations:** 1 School of Graduate Studies, Ariel University, Ariel, Israel; 2 Disaster Research Center, IL, Ariel University, Ariel, Israel; 3 Medical Call Centers, Natali, Ramat Gan, Israel; 4 Department of Health Systems Management, School of Health Sciences, Ariel University, Ariel, Israel; 5 Adelson Faculty of Medicine, Ariel University, Ariel, Israel; Max Stern Yezreel Valley College, ISRAEL

## Abstract

**Purpose:**

The COVID-19 pandemic has led to the isolation of the population in Israel, including the elderly. The present study aimed to compare the consumption of medical services among adults over the age of 65 in Israel at the time of the first COVID-19 lockdown relative to the corresponding period the year before.

**Methods:**

We conducted a retrospective longitudinal observational quantitative research based on the *Natali Healthcare Solutions Israel* database of subscribers. Company subscribers over the age of 65 (N = 103,955) were included in the sample (64.5% women) in two time periods, before the COVID-19 outbreak-P1, in 2019, and during the first COVID-19 lockdown- P2 in 2020. Logistic regression was applied to examine service consumption for study variables.

**Results:**

The average number of referrals to services was lower during the COVID-19 lockdown period (M = 0.3658, SD = 0.781) compared to the corresponding period in the previous year (M = 0.5402, SD = 0.935). The average number of ambulance orders, doctor home visits and service refusals were higher when compared to the same period in the previous year. During both time periods, women (P1- M = 0.5631, SD = 0.951; P2- M = 0.3846, SD = 0.800) required significantly more (*p <* .*000*) services than men (P1- M = 0.5114, SD = 0.910; P2- M = 0.3417, SD = 0.753). Older, widowed people, living in non-Jewish/mixed localities, or in average or below average socioeconomic status localities required relatively more services to those with opposite socio-demographic traits (*p <* .*000*).

**Summary and conclusions:**

In a large sample of elderly in Israel, findings indicate a decrease in referrals to medical care during the first COVID-19 lockdown period, yet an increase in ambulance orders, doctor visits and service refusals. Socio-demographic characteristics showed a similar effect in both time periods. The period of the first COVID-19 lockdown was characterized by a higher incidence of medical service refusals as compared to the equivalent period in the previous year.

## Introduction

The COVID-19 pandemic has caught the world population with insufficient readiness, especially with respect to the elderly who are particularly vulnerable to serious health consequences. Travel restrictions, including the prohibition of leaving homes, as well as the fear of contracting the disease, have made it difficult for the elderly who are chronically ill and forced to isolate in order to prevent exposure to disease effects, with the government’s goal being that this was done to protect them and other at-risk groups [1–4]. The elderly were instructed to work remotely as much as possible, to use delivery services or family members for food and medication purchases, and to avoid any unnecessary meetings. It was shown that this age group strictly adhered to the guidelines as compared to the general population [1, 4, 5].

A study conducted in the United States following the national emergency subsequent to COVID-19, has found that the elderly fear obtaining emergency room care, and a decrease of 72% was observed in the number of visits to emergency medicine clinics. Despite these data, an increase in visits to emergency medicine clinics was observed with respect to symptoms of infectious diseases or mental health problems [6]. Additional studies conducted in the United States have found similar findings which reinforced the fear of getting to the emergency room for medical care. Even patients with acute myocardial infarction—heart attack, arrived less to the emergency room, in a manner which delayed treatment of conditions that may result in mortality without appropriate treatment [7–9].

Technology has been the heart of adaptation in general and among the elderly population in particular. Telehealth services became the center of the medical field’s response, with elderly and high-risk patients staying home and being treated remotely [10]. In Israel, some of the response to elderly needs during the COVID-19 pandemic was given through technological means that assisted them in coping with the pandemic. These services ranged from medical to logistics services as well as personal telephone support [11]. Various socio-demographic aspects as age, gender and marital status that characterize the elderly population have been found to be related in their readiness to deal with a global catastrophic event such as the COVID-19 pandemic [12] The present study aimed to examine the effect of the COVID-19 pandemic on service consumption among the elderly in Israel during the COVID-19 lockdown in 2020 as compared to the corresponding period in 2019 by socio-demographic characteristics.

### Socio-demographic variables as risk factors in service consumption among the elderly

A person’s physical function is mainly based on a combination of age, illness and mobility by poor physical function including continuous decline, falls, poor mental health and increased mortality [13]. In addition, multiple morbidities, disabilities, and lack of functional independence have been shown to be risk factors for the adaptive daily conduct of the elderly [14, 15]. A previous study has found that loneliness and older age have been found to be risk factors for low adherence to guidelines, such that the older or lonelier a person is, the lower their compliance with guidelines [16]. Many seniors experience situations of loneliness, high morbidity, disability, lack of independence, multiple medication use and income struggles. These may lead to mental burnout that delay recovery from conditions they are experiencing, as well as a decrease in compliance with medical recommendations, a matter indicating disobedient behavior that can lead to various complications [16–18]. On the other hand, as people age, the higher the mortality rate is and as a result, the higher is the fear of mortality [19].

With respect to gender in old age, various studies have found that women tend to consume more medical services, adhere more with medical guidelines, attend more to medical examinations, and obtain emergency services in higher frequency as compared to men [11, 20–23].

Social support is identified as a strategy that can improve health outcomes [24]. Family members and social support within the family contribute greatly to the management of diseases among the elderly [25]. A person’s family status is found to be related to receiving support in times of crisis and are a crucial factor in disease management [24, 25]. A married person, or one in a relationship, is assisted by the partner who wakes up with them in the morning, is aware of their changing condition, reminds them to take their medications and the likes. Being without the support of a partner has a negative impact on the management of chronic illness and medical condition. An article by Liao and colleagues has found that mutual support between elderly spouses improves lifestyle and reduces complications among chronic patients, especially in the initial prevention phase of disease deterioration [26]. Older people were found to be more likely to require emergency services among married couples than among unmarried partners [27]. Older widowed women tend to ignore their deteriorating health, if they do not suffer from a serious medical condition [28]. There is a relationship between people adhering to values, beliefs and customs based on religion and healthy behavior in old age. It was found that the degree of religiosity influences a person’s decisions, their behavior, and their degree of compliance with medical guidelines. Often, and especially in old age, religiosity is a powerful source of spiritual strength, comfort, and hope, and has an impact on decision-making and medical compliance [29].

Health disparities between Jewish and Arab patients may stem, among other things, from cultural differences and accessibility to health centers. It had also been shown that the demand for emergency care is higher among adults than among young people and higher among Jews (3.1 times) than among Arabs (2.1 times) [20].

In a retrospective study based on 96,795 casualties from the Israeli National Trauma Registry, differences were found regarding the possibilities for injury among Jewish and Arab elderly populations, the severity of the injuries and the transportation to the hospital. Jews, relative to Arabs, were more likely to be hospitalized for more than seven days, admitted to the intensive care unit and to be discharged to a rehabilitation center. However, no differences were found with regard to mortality and surgery. The authors suggested that cultural differences and accessibility may partially explain these disparities [20]. This may be compared to the highest prevalence of COVID-19 in the USA being observed mainly among African American and Latino ethnic minorities, as well as among immigrants, as these groups are characterized by low socioeconomic status, inaccessibility to medical services, exacerbation of morbidity and complications of the disease, and are at increased risk for exposure to COVID-19 within their workplaces and in their more crowded living situations [30].

There are many differences in family relationships and composition. Among Arab nuclear families children and grandchildren are actively involved in the lives of their parents and grandparents, the elderly live with family members, elderly Arabs prefer home care rather than rehabilitation. In Israel, there is a state health service law for the entire population, but the accessibility of clinics and ambulances is lower in the Arab population due to the fact that they live relatively more in the periphery [20].

The previous pandemic which led to closures in different continents around the world, occurred about 100 years ago during the Spanish influenza outbreak [31]. The relationship between socio-demographic characteristics of the elderly population in the context of consumption of medical services has not been sufficiently investigated and there are not sufficiently relevant publications examining this topic since that Spanish influenza outbreak. The importance of examining these characteristics among Israel’s elderly population at the time of the first lockdown and its implications to other lockdowns during the pandemic is significant for the purpose of understanding the needs of this population with respect to health promotion, especially when this part of the population has been continuously growing in percentage throughout the years. The purpose of the present study was to examine the variability in demand of medical services among elderly in Israel during the first COVID-19 lockdown in comparison to the corresponding period in the previous year, by distribution of various socio-demographic variables, as well as how the demand for medical services is characterized according to different socio-demographic variables: gender, age, marital status, ethnicity, degree of religiosity and socioeconomic status. We hypothesized that service consumption would be lower during the COVID-19 pandemic compared to the corresponding period in the previous year, and that men, widowers, adults in the younger age group as well as non-Jews, will consume fewer services in comparison to women, married, older individuals and Jews.

This paper will also examine if refusal to services of Natali’s elderly subscribers was higher during the pandemic closure.

## Methods

This study is a longitudinal observational retrospective study that compares the consumption of medical services by the elderly in two time periods. The first period–prior to the start of the pandemic in February-April 2019 (P1). The second period—the corresponding months in the following year, February-April 2020 (P2), in which the citizens of Israel were subjected to the COVID-19 lockdown restrictions imposed by the government, in accordance with the Ministry of Health directives and guidelines.

The study is based on a database from *Natali Healthcare Solutions in Israel*, which is a private company for the provision of medical services that has been operating in Israel for over 30 years. The company provides a wide range of medical and non-medical services to elderly subscribers and is part of a range of organizations operating in the field medical service provision to Israeli residents. The company granted 1.1 million interactions and services to the population of Israel that contains 9.444 million people- 11.6% from all Israeli population. The company’s services include telemedicine services supported by call center representatives who have been trained as emergency call medics, as well as medical consultation calls with various doctor, emergency medical services such as ambulance services and doctor home visit services. As part of the service, the company provides subscribers with medical devices such as: distress buttons, (Electrocardiogram) ECG devices, various sensors, and passive monitoring devices [32].

### Population

The study population is adults over the age of 65 who in Israel who are *Natali Healthcare Solutions in Israel* subscribers. The subjects in this study were not sampled, but all of *Natali Healthcare Solutions* company members over the age of 65 were included in the datafile. Of the full subscriber list for the years 2019–2020, the following customers who did not meet the study criteria were removed: those under the age of 65 (in order to focus only on elderly), those whose services were terminated for various reasons (cancellation, death, service freeze—2% of the sample- in order to keep only those who had data on both periods), those whose number of calls to the center was over 400 inquiries in both periods (n = 17; probably caused by logistical malfunctions of emergency button).

### Study variables

#### Dependent variables

Various medical needs constituted the dependent variables in the study. The dependent variables were measured, for each period separately: before COVID-19 (P1), and during lockdown (P2). Table 1 describes the dependent variables in this study.

**Table 1:** The dependent variables description.

	Dependent variables	definition of the variables	*P1	*P2
**1**	**P1/P2 Medical calls to the call-center [33]**	The number of times a subscriber contacted the call center for medical reasons	0–114	0–472
**2**	**P1/P2 Emergency calls to the call-center**	The number of times a subscriber contacted the emergency call-center	0–226	0–294
**3**	**P1/P2 Ambulance orders [34]**	The number of times the subscriber ordered an ambulance during these periods	0–18	0–16
**4**	**P1/P2 Doctor home visits [35, 36]**	The number of times a subscriber ordered home visit by a doctor during these periods	0–102	0–111
**5**	**P1/P2 Total referrals index (variables 1+2)**	Count of medical referrals and emergency referrals together for each subject	0–294	0–540
**6**	**P1/P2 Service consumption (variables 1–4)**	Variables 1–4 dichotomized and combined	0–4	0–4
			α = 0.693	α = 0.630
**7**	**P1/P2 Ambulance cancellations**	The number of times the subscriber canceled an ambulance ordered during these periods	0–7	0–6
**8**	**P1/P2 Service refusal**	The number of times during the period, in which an ambulance service or a doctor visit were offered by the call center, but the subscriber refused the service	0–6	0–18

*Data under columns P1 and P2 represent the ranges for each variable in each period.

#### Independent variables

The data file contained the socio-demographic variables of the subjects, which were used as independent variables in this study as detailed: Gender—male / female. Age—calculated at the starting point of the study—2019 by year of birth. The age of two of the subjects was unknown. A new age variable was created by grouping the ages into three categories [37] as detailed: young seniors (65–75), middle-aged adults (76–85), older adults (86 and older). Marital status—single / married / divorced / widowed. 28,742 subjects (27.5% of the sample) were missing data on marital status.

Three additional socio-demographic variables were derived from the respondent’s residential address after cross-referencing the information with Israel Central Bureau of Statistics (CBS) data [37] as follows: Ethnicity, degree of religiosity and the socio-economic status of the residence. For ethnicity of the residence–the residential address of the subscriber was examined via CBS database. The values of the variable are: 1. Jewish locality, 2. non-Jewish locality, and 3. mixed ethnic locality. The degree of religiosity of the residence was derived by city of residence. The values of the variable are: 1. a secular locality, 2. a religious locality, and 3. ultra-Orthodox locality. Socio-economic status of the residence—according to CBS coding, the localities are classified into clusters from 1–10, with 1 symbolizing a very low socioeconomic status, and 10 a very high socioeconomic status. This scale was divided into three categories: 1. Low socioeconomic status—clusters 1–5, 2. Middle socioeconomic status—clusters 6–7, and 3. High socioeconomic status—clusters 8–10 [38]. For the purpose of logistic regression, we dichotomized these values into 2 groups: 1. Low and high SES; 2. Average SES.

### Data analysis

For data analysis purposes, the data in Table 2 presents descriptive statistics expressed in percentages and count. All missing values were omitted from the description of the variables in Table 2, which displays the valid percent value as well as the raw number value of the subjects in each category. Table 3 presents averages and standard deviations using a T test for paired variables comparing between the two periods. The 2-tailed significance of the T value analysis is presented. In order to examine whether there were any differences between needs in P1 and P2 within study groups we performed mixed repeated measures. Table 4 presents the averages of the index comprised of “Service consumption” by period. A Two-way mixed ANOVA with Bonferroni correction for pairwise comparisons, was used to examine the differences by group, time, and by the interaction between group and time while examining significance by Bonferroni. Similarly, Table 5 presents the same analysis for the dependent variable of service refusal. Values displayed in Bold—are those significantly higher values than the other groups. Table 6 presents logistic regression results for predicting service consumption according to the study’s socio-demographic variables. For this purpose, the dependent variable "Service consumption" was divided dichotomously in P2 by the median. The β values displayed in the table constitute the Relative risk—RR and present the probability of Service consumption according to the independent variable groups. The confidence Interval–(CI) Range displays the minimum and maximum values for the range with a 95% of confidence. Values displayed in Bold—are significantly higher values than the other groups.

**Table 2:** Sample description by socio-demographic variables.

Variable	Values	Total Sample	
		n	%
**Total Sample**		**103,955**	**100%**
**Gender**	Males	65,954	63.5
	Females	36,364	35.0
	Unknown	1,637	1.6
**Age categories**	Young seniors (65–75)	30,252	29.1
	Mid-age seniors (76–85)	47,846	46.0
	Elder seniors (86 and above)	25,855	24.9
**Marital status**	Bachelor	1,105	1.5
	Married	46,360	61.6
	Divorced	4,496	6.0
	Widowed	23,252	30.9
**Ethnicity**	Jewish	87,210	83.9
	Non-Jewish	918	0.9
	Mixed	15,825	15.2
**Residential religiosity**	Secular residence	102,305	98.4
	Religious residence	421	0.4
	Orthodox residence	1,229	1.2
**Socioeconomic status of residence**	Below average (clusters 1–5)	39,124	37.6
	Average (clusters 6–7)	38,677	37.2
	Above average (clusters 8–10)	26,152	25.2

**Table 3:** Mean of service consumption for the elderly population. According to study periods–pre COVID-19 (P1) and during COVID-19 lockdown (P2).

		P1	P2	Significance (2 tailed)	T value
**Total referrals index**	M	1.194	0.756	0.000	32.86
	SD	3.71	4.32		
**Ambulance orders**	M	0.082	0.087	0.011	-2.55
	SD	0.38	0.40		
**Ambulance cancellations**	M	0.006	0.009	0.000	-8.10
	SD	0.08	0.10		
**Doctor home visits**	M	0.106	0.122	0.000	-7.25
	SD	0.60	0.72		
**Service refusal**	M	0.013	0.022	0.000	-12.22
	SD	0.14	0.20		

M = Mean, SD = Standard deviation, N = 103,955.

**Table 4:** Mean of medical service consumption among the elderly population in the pre-COVID-19 period (P1), and during COVID-19 lockdown (P2) by socio-demographic variables.

Variables / Values		P1			P2			Time sig.	Inter. sig.	N
		M	SD	Sig.	M	SD	Sig.			
**Gender**	Females	**.5631** ^**a**^	.951	0.000	**.3846** ^**a**^	.800	0.000	0.000	NS	65,954
	Males	.5114^b^	.910		.3417^b^	.753				36,364
**Age categories**	Young seniors	.3848^c^	.796	0.000	.2389^c^	.640	0.000	0.000	>0.001	30,252
	Mid-age seniors	.5500^b^	.933		.3734^b^	.776				47,846
	Elder seniors	**.7040** ^**a**^	1.052		**.5001** ^**a**^	.906				25,855
**Marital status**	Bachelor	.5946^b^	.931	0.000	.4027^b^	.796	0.000	>0.001	>0.001	1,105
	Married	.4571^c^	.884		.2978^c^	.711				46,360
	Divorced	.6210^b^	.953		.4446^b^	.826				4,496
	Widowed	**.7451** ^**a**^	1.013		.**5406**^**a**^	.906				23,252
**Ethnicity**	Jewish	.5355^b^	.932	0.000	.3630^b^	.779	0.000	>0.001	0.052	87,210
	Non-Jewish	**.6111** ^**a**^	.994		**.3725** ^**a**^	.741				918
	Mixed	**.5620** ^**a**^	.945		**.3808** ^**a**^	.795				15,825
**Residential religiosity**	Secular residence	.5398^a^	.934	0.097	.3659^a^	.781	0.109	>0.001	NS	102,092
	Religious residence	.4991^a^	.852		.3026^a^	.718				575
	Orthodox residence	.5893^a^	1.00		.3835^a^	.809				1,288
**Residential SES**	Below average	**.5547** ^**a**^	.945	0.000	.**3729**^**a**^	.784	0.000	0.000	NS	39,124
	Average	**.5575** ^**a**^	.941		**.3867** ^**a**^	.798				38,677
	Above average	.4929^b^	.909		.3241^b^	.748				26,152
**Total service consumption**		.5402	.935	0.000	.3658	.781	0.000		-	103,955

P1 = pre-COVID-19 period; P2 = during COVID-19 lockdown; M = Mean; SD = Standard deviation; Sig = Significant; Inter. Sig. = Interaction significant; N = Number of subjects; SES = Socioeconomic status; NS = Not significant. Significance testing for differences between groups a ≠ b ≠ c with **bolded numbers** signifies significantly higher values, *p <0*.*05*.

**Table 5:** Mean service refusal (ambulance and doctor visit) among the elderly population in the pre-COVID-19 period (P1) and during COVID-19 lockdown (P2) by socio-demographic variables.

Variables / Values		P1			P2			Time sig.	Inter. sig.	N
		M	SD	Sig.	M	SD	Sig.			
**Gender**	Males	**.0156** ^**a**^	.152	0.000	**.0248** ^**a**^	.219	0.000	>0.001	NS	65,954
	Females	.0102^b^	.118		.0185^b^	.165				36,364
**Age categories**	Young seniors	.0097^c^	.125	0.000	.0193^c^	.221	0.000	0.000	NS	30,252
	Mid-age seniors	.0137^b^	.140		.0220^b^	.190				47,846
	Elder seniors	**.0178** ^**a**^	.157		**.0265** ^**a**^	.195				25,855
**Marital status**	Bachelor	**.0244** ^**a**^		0.000	**.0281** ^**a**^	.208	0.000	0.000	NS	1,105
	Married	.0111^b^			.0187^b^	.189				46,360
	Divorced	**.0178** ^**a**^			**.0269** ^**a**^	.199				4,496
	Widowed	**.0186** ^**a**^			**.0299** ^**a**^	.229				23,252
**Ethnicity**	Jewish	.0136^a^	.141	0.867	.0224^a^	.196	0.758	0.003	NS	87,210
	Non-Jewish	.0120^a^	.127		.0174^a^	.160				918
	Mixed	.0131^a^	.138		.0224^a^	.229				15,825
**Residential religiosity**	Secular residence	.0135^a^	.140	0.212	.0224^a^	.201	0.932	0.019	NS	102,092
	Religious residence	.0052^a^	.072		.0191^a^	.171				575
	Orthodox residence	.0186^a^	.170		.0233^a^	.174				1,288
**Residential SES**	Below average	.0136^a^	.138	0.295	.0215^b^	.194	0.013	0.000	NS	39,124
	Average	.0142^a^	.143		**.0246** ^**a**^	.221				38,677
	Above average	.0125^a^	.139		.0202^b^	.178				26,152

P1 = pre-COVID-19 period; P2 = during COVID-19 lockdown; M = Mean; SD = Standard deviation; Sig = Significant; NS = Not significant; Inter. Sig. = Interaction significant; N = Number of subjects; SES = Socioeconomic status.

Significance testing for differences between groups a ≠ b ≠ c ≠ d with **bolded numbers** signify significantly higher mean times of refusal, *p <0*.*05*.

**Table 6:** Logistic regression results for the prediction of service consumption during COVID-19 lockdown by socio-demographic variables and pre-COVID-19 service consumption.

Variables	Values	RR	Confidence interval (CI)	
			Low	High
**Gender**	0 = Females, 1 = Males	**1.093** ***	1.049	1.138
**Age**	0 = Younger, 1 = Elder	**1.397** ***	1.340	1.457
**Marital status**	0 = Married, 1 = Not married	**1.757** ***	1.688	1.828
**Ethnicity**	0 = Jews, 1 = Non-Jews	1.001	.984	1.018
**Residential religiosity**	0 = Secular, 1 = Religion	1.001	.922	1.085
**Residential SES**	0 = Low and above average, 1 = Average	**1.137** ***	1.095	1.181
**P1 Service consumption**	0 = No consumption, 1 = Yes	**2.489** ***	2.441	2.537
**n**				**74,878**
**Explained variance**	**Nagelkerke R Square**			**24.0%**

*p < .05,

**p < .01,

***p < .001, RR = Relative risk.

The study was approved by the Ariel University institutional ethics committee (Approval No. AU-HEA-AZ-20200624) and has also received the approval of *Natali Healthcare Solutions in Israel* management to conduct the research. Data from the research file was taken retrospectively and anonymously from the company’s database without identifying details of the subjects. The subjects’ records were kept in an encoded file in a manner which ensured the identification of the subjects stayed concealed and that no link could be made between the data and the individuals sampled.

## Findings

The data file containing 103,955 records of *Natali Healthcare Solutions in Israel* subscribers in two periods was the platform for analysis that examined the research questions.

Table 2 presents the final sample according to the socio-demographic variables examined.

A total of 103,955 *Natali Healthcare Solutions in Israel* subscribers participated in the study, representing the entire customer population for the 65+ age group (Mean age 80, SD = 7.46, Range 65–119). Table 2 shows that the sample included 64.5% women (n = 65,954) and 35.5% men (n = 36,364). Gender information was missing for 1,637 participants. The average age of the subjects was 80 years (SD = 7.46) which we divided into three age categories: 29.1% constituted the young age group- 65–75, 46.0% constituted the middle age group- 76–85, and the oldest age group of those aged 86 and over accounted for 24.9% of the sample. Regarding marital status- 61.6% of the sample were married and 30.9% widowed. The sample had a Jewish 83.9% and secular 98.4% majority. Over one third of the sample (37.6%) belong to settlements below average SES (Socioeconomic status of place of residence), 37.2% on the average and around a quarter is above average (25.2%) according to CBS coding.

First, we examined service consumption including the total referrals index, ambulance orders, ambulance cancellations, doctor home visits and the refusal of service. Table 3 displays the differences between the dependent variables in the two time periods.

It can be seen from Table 3 that the averages of all types of service consumption differed between the pre-COVID-19 period (P1) and the COVID-19 lockdown period (P2) significantly. Total referral index were the only variable that averaged higher in P1 (M = 1.194, SD = 3.71) in comparison to P2 (M = 0.756, SD = 4.32) (*p <* .*000*). That is, during P2, there was a lower average of calls to the center relative to P1. Ambulance orders (M = 0.087, SD = 0.40) and ambulance cancellations (M = 0.009, SD = 0.10) were higher in P2 than in P1 (M = 0.082, SD = 0.38; M = 0.006, SD = 0.08). Similarly, the average number or ordering a doctor home visit was higher during first COVID-19 lockdown period (M = 0.122, SD = 0.72) than during the pre-COVID-19 period (M = 0.106, SD = 0.60). Also, refusal of service (ambulance or doctor visit) was higher in P2 (M = 0.022, SD = 0.20) than in P1 (M = 0.013, SD = 0.14).

Table 4 presents the averages of the collective index of service consumption, which was consisted of medical calls to the center, emergency calls to the center, ambulance orders and home doctor visits pre-COVID-19 and during the first COVID-19 lockdown period and according to various socio-demographic variables.

It can be seen from Table 4 that in total, the average demand for services decreased from the pre-COVID-19 period (M = 0.5402, SD = 0.935) in relation to the first COVID-19 lockdown period (M = 0.3658, SD = 0.781) significantly between the two periods. In both time periods, females (P1-M = 0.5631, SD = 0.951; P2- M = 0.3846, SD = 0.800) required more services than males (P1- M = 0.5114, SD = 0.910; P2- M = 0.3417, SD = 0.753) (*p <* .*000*). In both time periods, the older age group required more services than the younger age groups, widowers required more services in relation to single, divorced or married individuals; subscribers residing in non-Jewish or mixed localities required more services in relation to those living in Jewish localities; and seniors living in localities socioeconomically rated as average or below average required more services in relation to those living in above average rated localities (*p <* .*000*). No significant differences in the request for services were found according to the degree of religiosity of the locality in the two time periods.

Similarly to what is shown in Tables 4 and 5 shows the average of ambulance or home doctor visits, service refusals, pre-COVID-19 and during the first COVID-19 lockdown period and according to various socio-demographic variables.

It can be seen from Table 5 that in both time periods, males (P1- M = 0.0156, SD = 0.152; P2- M = 0.0248, SD = 0.219) refused service more than females (P1- M = 0.0102, SD = 0.118; P2- M = 0.0185, SD = 0.165) (*p <* .*000*). In both periods the older age group refused service more than the younger age groups, and unmarried seniors- single, divorced and widowers refused service more than married seniors significantly. No significant differences were found in the refusal of services by sector or degree of religiosity by locality in the two periods. In P2 the average service cancellation was highest among localities ranked in the average socioeconomic status (M = 0.0246, SD = 0.221) relative to low socioeconomic status localities (M = 0.0215, SD = 0.194) or above average ones (M = 0.0202, SD = 0.178). All differences presented in Table 5 were significant for time, but no interaction was detected.

Table 6 presents results of logistical regression for predicting service consumption during the first COVID-19 lockdown period from socio-demographic variables and pre-COVID-19 service consumption.

The logistic regression analysis shows that the most powerful variables for predicting service consumption during P2, are demand for services in the previous year (RR = 2.489, p < .000, 95% CI- 2.441–2.537), marital status (RR = 1.757, p < .000, 95% CI-1.688–1.828), and age (RR = 1.397, p < .000, 95% CI-1.340–1.457). The variables of gender and socioeconomic status came out significant but not as strong in the model. The variables of sector and religiosity did not come out significant in the model. These variables explain 24.0% of the variance of the dependent variable–service consumption during the COVID-19 lockdown period of time. These findings indicate that the chance of requiring service at the time of the first COVID-19 lockdown was 2.5 times higher among those who required services in the previous year: 1.7 times higher among unmarried seniors, and 1.4 times higher among the older age group of 85 and over in comparison to the younger seniors.

## Discussion

The COVID-19 pandemic has greatly affected the elderly population [1], and the various restrictions imposed due to it have resulted in refraining from leaving the home for various needs and even for the purpose of receiving medical care [1, 3]. This study characterized the consumption of services among the elderly in Israel during the first COVID-19 lockdown in 2020 compared to the corresponding period in 2019, according to various socio-demographic characteristics, based on data from *Natali Healthcare Solutions Israel* database of subscribers. This study is unique both in examining topics that have not come up in previous Israeli studies examining the COVID-19 effects, as well as in its reliance on a very large sample of the elderly population in Israel, which requires medical resources and social support in routine times. Consumption of the various services that *Natali Healthcare Solutions Israel* offers, was examined in this study with reference to inquiries made to the call center–via phone or distress button, ambulance orders, doctor home visits as well as refusals to receive service. These variables were analyzed in both time periods while examining their distribution by gender, age, religious sector, degree of religiosity and socio-economic status.

Our findings show that during the period of the first COVID-19 lockdown in 2020, the average number of service consumption by elders was significantly lower as compared to the corresponding period in 2019, in line with the study hypothesis. During the pandemic, consumption of medical services was expected to decrease, as found in a study conducted in the USA in which chronic patients turned less often to medical service centers [6]. It was found that people avoided going to emergency rooms; The elderly’s calls to the center during the COVID-19 period decreased even when experiencing life-threatening situations, [6–9]. On the other hand, the average number of ambulances calls, and doctor home visits increased. This might be explained as the elderly didn’t want to go to the Emergency room (ER) and to be exposed to other patients that may had COVID-19. Doctor visits and ambulance services provide medical services at home, and often decrease ER visits and hospitalization. Findings show that during the COVID-19 period, the elderly population had higher rates of refusal for services. Consuming services and refusing them are different phenomena. They have different meanings and different importance, although both actually express concerns from the situation, and could explain the fear that might existed among elderly population. Refusal signifies a situation in which a person received advice and a recommendation to receive medical treatment that he decided not to receive against the medical advice. The fact that there is an increase in refusals similarly suggests there might be serious fear of infection in this population.

It is possible that the lower average of service consumption during the first COVID-19 lockdown, as found in the present study, is due to an increase in the anxiety levels and fears of the elderly about contracting diseases due to the higher likelihood of dying from it [34] and due to this concern they ordered less medical services during this period. It may also be that elderly people, decided not to consume the medical services they know and are insured for, as to not burden the medical staff who were stretched thin, as well as their fear of being exposed to those routinely working with sick people. This phenomenon is observed in times of crisis when people who are in distress avoid calling security services and try to fend for themselves [7–9].

Older people require more medical services than young people [9]. The current study also found that the average service consumption among older adults over the age of 85 is higher. This is also the age group that refused more of the services. Moreover, the widowed subscribers consumed on average more services than single and divorced individuals, and significantly more than married ones. In this study, marital status was found to be a substantial and significant predictor for medical services demand, with the likelihood of unmarried people to require more medical services being higher, as is supported by the literature [24–26]. In our study we found significant time and group interactions for age categories. These significant interactions might be due to different behaviors of the groups over time.

When examining the literature to explore consumption of medical services by gender, it is shown that women more than men, tend to consume more medical services, adhere more to medical guidelines, obtain more of the required tests, as well as arrive more at the emergency room for medical treatments [16, 21, 22]. The subjects of this study constitute the population of Natali Healthcare Solution which do not represent the gender ratio in Israeli population. Women’s rates in the Israeli population at this age are higher than man. Men are considered complex patients at this age and it is reasonable they consume more medical services. The decrease that occurred in the demand for services during the COVID-19 lockdown, pertains to both sexes. Future research should deal more in depth with women versus men differences.

Ethnic gaps between Jews and Arabs may stem, among other things, both from cultural differences as well as from accessibility to health centers [20]. For example, elderly Arabs live with family members and prefer home care rather than rehabilitation [31] but also have less accessibility for clinics and community medical services. In the USA, higher rates of COVID-19 morbidity have been observed among ethnic minorities, whom have less access to medical services and health promotion abilities and have a lower socio-economic status relative to the general population. Minorities have less access to medical care and have a higher incidence of underlying morbidity leading to more serious disease [30]. This contrasts our study data which shows that subscribers living in non-Jewish or mixed localities consumed on average more services than subscribers living in Jewish localities. The lack of accessibility to clinics and medical services in the community might explain the increase in demand for medical services at home. Perhaps this is different in Israel where there is public health system to all and the demand for services is more accessible to vulnerable populations than in the USA. It may be that to the Arab minority in Israel it took longer to understand the Ministry of Health’s restrictions in the first closure. It should also be noted that the ethnicity variable was also based on residence data and not on self-report data and hence may not be accurate. In addition, subscribers living in localities ranked in a lower than average or average socio-economic status according to the CBS [39] consumed more medical services compared to subscribers living in localities ranked in above-average socioeconomic status localities.

The logistic regression findings show that the most significant predictor of service demand during COVID-19 is service demand during the previous year. The chance of service demand during the first COVID-19 lockdown period is approximately 2.5 times higher among those who required service in the previous year. This demonstrates the greater need that exists among at-risk populations, which require emergency medical services even during routine times.

Medical services consumption has been decreased during COVID-19. The COVID-19 pandemic has significantly increased the need for remote monitoring and treatment of patients. That could be intensified by different reasons: lack of convenience of remote treatment; transportation issues; patients’ fear of coming into contact with medical staff during the pandemic and has revolutionized the health care system in the field of telemedicine. An example for increased fear was given with the experience of depression and anxiety among elderly who were unable to obtain medication were reported during the COVID-19 [40, 41]. Telemedicine kits are used among *Natali Healthcare Solutions in Israel* to remotely monitor subscribers in a way that completely changes the medical approach preceding the pandemic outbreak [10]. One explanation for that could be that because telemedicine developed quickly, and the company is using healthcare technological solutions, seniors might prefer to receive essential medical care during the COVID-19 pandemic. Needs of the elderly must be addressed even when health services are under pressure due to an epidemic outbreak. Policy makers must take care of the health needs of people whose vulnerability to an epidemic is increases [42].

The very large sample of adults in Israel who consume medical services during routine times is one of the main strengths of the present study. The data file of *Natali Healthcare Solutions in Israel*, a leading private company in Israel for providing medical care to the elderly, was analyzed here for the first time. Another strength of the study is its being a longitudinal study, based on the same subjects in both time periods, which contributes to the strength its findings.

### Study limitations

A major limitation of this study is derived from its strength as it takes the whole subscribes population at elderly age. But, doing so, created a bias of the natural population at this age in Israel. The sample is actually the population of the company’s subscribers at this age and does not pretend to represent the population of adults in Israel. Readers should be aware of gender differences that may result differently than expected. Part of the study limitations is the lack of information pertaining to additional data such as the degree of religiosity, socio-economic status, and ethnicity, which could not be unequivocally examined since the data was based solely on place of residence, and not based on self-report. Another study limitation is that potentially interfering variables such as disease severity, number of diseases, mental state, and nutritional balance, were not taken into account in this study, given the unavailability of this data.

## Conclusions

The findings of the study show a decrease in referrals to medical centers during the first COVID-19 lockdown period, an increase in demands for medical services such as ambulance orders and doctor home visits, and an increase in service refusals. No significant differences were found in trends of socio-demographic characteristics over time. The pandemic period raised concerns among the elderly and was characterized by a higher incidence of medical service refusals.

Consumption of services among the elderly during first lockdown of COVID-19 decreased compared to the previous year. Future studies should lead policymakers to better respond to the needs of this population in times of a health crises.

In summary, this manuscript explores the critical issues of health care access and use by the elderly population during the COVID pandemic. The advantage of this analysis stem from using a retrospective cohort of a large population and a scale to measure health care consumption, capturing the multifaceted nature of this response. These findings constitute a critical initial step towards eliciting health care use behaviors among the elderly during COVID and similar crises. There were no significant differences between the periods according to different socio-demographic characteristics. The results shows that health care consumption increased in this population between the pre- and post-COVID, particularly among specific sub-groups like older clients, the widowed, non-jews or mixed ethnicities, and those at or below average SES. Females tend to consume more services than males. The chance of requiring medical service at the time of the first COVID-19 lockdown was higher among those who required services in a previous year, among unmarried seniors, and among the older age group of 85 and over in comparison to the younger seniors. The implications of these findings reinforce the need for social response for the elderly in order to improve their quality of life, especially during a pandemic period in which adult life is at risk. Inter-institutional cooperation is required to produce good social programs for the preservation of the quality of lives of the elderly.
